# An Update of the Interstitial Cell Compartment in the Normal Human Bladder

**DOI:** 10.1155/2014/464217

**Published:** 2014-02-27

**Authors:** Kamiel A. J. Kuijpers, John P. F. A. Heesakkers, Theo G. M. Hafmans, Jack A. Schalken

**Affiliations:** ^1^Department of Urology, Radboud University Nijmegen Medical Centre, 267 Nijmegen, The Netherlands; ^2^Department of Biochemistry, Radboud University Nijmegen Medical Centre, 267 Nijmegen, The Netherlands

## Abstract

*Aims*. Interstitial cells, also called myofibroblasts, most probably play a major role in the pathogenesis of the overactive bladder. However, no specific phenotypic marker has been identified. We investigated whether N-cadherin could play a role as a discriminatory marker for interstitial cells in the human bladder. *Methods*. Bladder biopsies (*n* = 16) were collected from macroscopically nonpathological locations during cystectomy which was performed because of bladder cancer. Tissue was analyzed for expression of N-cadherin. N-cadherin+ cells were phenotyped using antibodies against PGP9.5, smoothelin, vimentin, and C-kit. Findings were related to bladder tissue histology and ultrastructure of myofibroblastic cells. *Results*. N-cadherin+/vimentin+ cells with branched cell bodies were found in the lamina propria and detrusor layer. They were closely associated with neurons and showed no colocalization of PGP9.5 or smoothelin. A second type of N-cadherin+ cells was found at the boundary of detrusor bundles and in the lamina propria. These cells colocalization C-kit. We assumed that N-cadherin+/vimentin+ cells are similar to the ultrastructurally defined myofibroblasts. *Conclusions*. N-cadherin can play a role as a discriminatory marker for interstitial cells in the human bladder, as the interstitial compartment of the human bladder houses a population of cells from mesenchymal origin, immunopositive for N-cadherin, vimentin, and C-kit.

## 1. Introduction

The human bladder shows spontaneous contractile activity during the filling phase of the micturition cycle [1]. As this activity is also found in bladders that are isolated from the central nervous system, it seems that it is generated within the bladder wall. The overactive bladder (OAB) syndrome is associated with complaints of frequency and urgency that typically occur during the filling phase [2, 3]. Spontaneous contractile activity of the bladder may share characteristics with peristaltic activity in the gastrointestinal tract [4]. Specialized pacemaker cells of the gut, also known as interstitial cells of Cajal (ICCs), are thought to behave as pacemaker cells that transmit their electrical activity to the smooth muscle [5, 6]. Interstitial cells (ICs) are also found in the human bladder [7, 8]. These cells are located throughout the lamina propria and detrusor layer. It has been suggested that they form a network integrating signals and responses in the bladder wall between various types of cells. However, ICs have several subtypes based on morphological appearance and differential expression of markers, like myofibroblasts, making the search for their exact functional role in the human bladder a contentious subject.

Recent studies have identified various surrogate histochemical markers for ICs, such as the stem cell receptor C-kit and cyclic guanosine monophosphate (cGMP) [8]. However, the specific immunophenotype of ICs is still controversial. C-kit is not expressed by all types of ICs and it can also be detected in other cell types [9]. Additionally, many C-kit antibodies fail to detect ICs in positive control tissues. cGMP is a marker for ICs in the bladder but it is also expressed by urothelial cells [8]. Thus, so far, no marker has been identified that can be considered as a specific phenotypic marker for ICs in the human bladder. Therefore, irrefutable confirmation of the interstitial phenotype still depends on application of transmission electron microscopy (TEM), which is highly time consuming.

Cadherins constitute a superfamily of glycoproteins that participate in cell-cell recognition by functioning as signaling centers [10, 11]. We have previously shown that cadherin-11 is expressed by ICs in the lamina propria [12]. Cadherins may therefore play a role in regulating an intramural network of these cells. As subpopulations of bladder ICs exist, another subclass of cadherins might account for a specific discriminatory marker for interstitial cells in the interfascicular planes of the detrusor layer. N-cadherin is known to regulate mesenchymal cell development [13] and is the most commonly expressed cadherin in stromal cells [14, 15]. We therefore investigated the expression of N-cadherin in the normal human bladder. We used additional immunohistochemical cell markers as well as transmission electron microscopy (TEM).

## 2. Material and Methods

### 2.1. Patients

Bladder biopsies (*n* = 16) were collected from sixteen individuals in whom radical cystectomy was performed because of muscle invasive bladder cancer. Mean patient age was 62 years (52–75), nine males and seven females. Samples were dissected from tumor-free bladder areas at least 3 cm distant from tumor zones. Biopsies were all taken from the vesical dome from functionally stable bladders. All of the patients underwent primary resection in terms of early cystectomy and none of the patients underwent intravesical installation. The local ethics committee approved the study and informed consent was obtained from all patients. Full thickness specimens were collected and placed in a mould containing Tissue-Tek (Sakura) for cryosectioning. Specimens were snap frozen in isopentane at –80°C. Tissue was checked for intact urothelium using a hematoxylin-eosin stain.

### 2.2. Immunohistochemistry

Sections of 4 *μ*m specimens were prepared using a cryostat and mounted on Super Frost Plus slides (Menzel-Gläser). The unfixed sections were immersed in 3% paraformaldehyde for ten minutes and stained for N-cadherin (M142 Takara; C2542 Clone GC-4 Sigma). Cell membranes were permeabilized in 0.2% Triton X-100 for 5 minutes. For cytoskeletal protein staining, samples were fixed in acetone for ten minutes and air dried at room temperature for 2 hours. Each step was separated by wash in magnesium and calcium containing PBS (PBS-Extra: 40 mL 25x PBS, 960 mL demi-water, 100 *μ*L 1 M MgCl_2_, 100 *μ*L 1 M CaCl_2_). Sections were incubated for 1 hour using primary antibodies diluted in PBS 1% bovine serum albumin for blocking. Sections again were washed three times in PBS-Extra. Next, the sections were incubated with Alexa Fluor 488 (A-11017, A-11070 Molecular Probes) or Alexa Fluor 594 (A-20185, A-11032 Molecular Probes). Finally, treatment with DAPI (24653 Merck) was performed for staining the nuclei. All sections were mounted in Fluorescent Mounting Medium (S3023 Dako Cytomation). Negative controls included omission of primary antibodies. The following antibodies were used to further phenotype N-cadherin+ cells: PGP9.5 (a pan-neuronal marker) (7863-0504 AbD Serotec), smoothelin (specific marker for smooth muscle cells [16]) (R4A ab8969 Abcam), vimentin (marker for fibroblasts) (RV203 Eurogentec), and C-kit (CD117 DAKO). For the latter antibody, specimens of human jejunum were used as positive controls.

### 2.3. Transmission Electron Microscopy

Sixteen human normal bladder biopsies were also processed for standard transmission electron microscopy (TEM). Processing for TEM was done according to the standard protocol using Somogyi fixative [17]. Ultrathin sections were photographed using a TEM 1010 electron microscope (JEOL, Peabody, Massachusetts).

### 2.4. Analysis

Immunostained sections were examined by binocular epifluorescent microscopy (Leica DFC FX). Four times ten slides were analyzed per full-thickness specimen. Each set of ten slides was separated by approximately 5 mm of tissue. Cryosections were also stained with hematoxylin-eosin to interpret the fluorescent images. Morphology, phenotypic expression of above mentioned markers, and the ultrastructure of myofibroblastic cells were evaluated.

## 3. Results

### 3.1. N-Cadherin Expression in Normal Human Bladder

**Figure 1:**
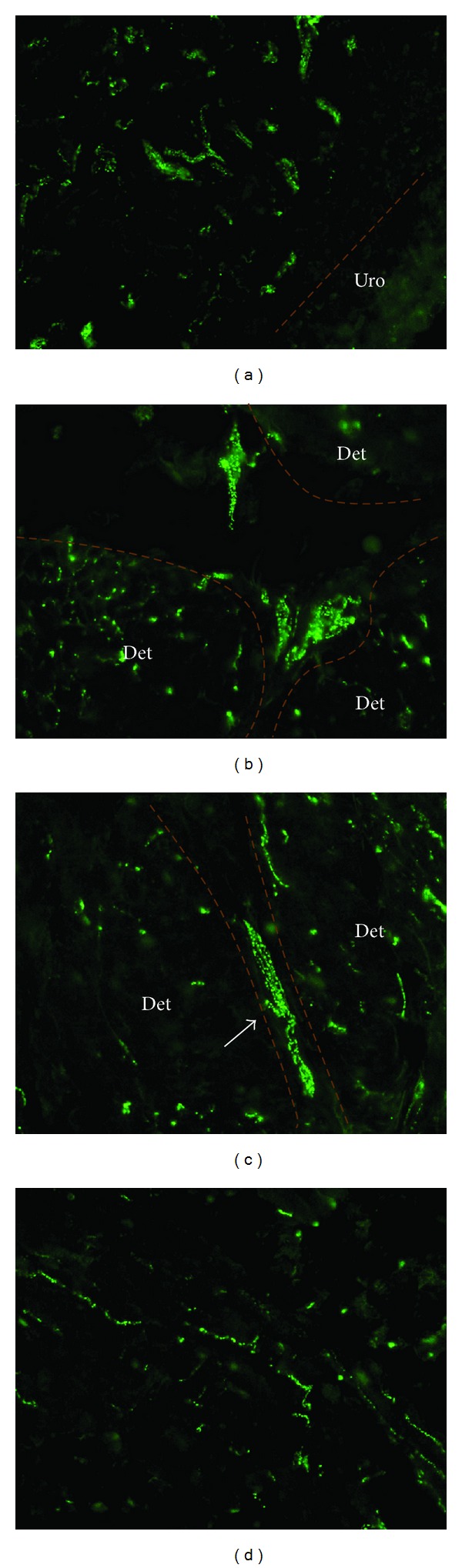
N-cadherin+ structures in the normal bladder wall. (a–d) A punctate signal for N-cadherin (green) reveals numerous positive N-cadherin+ cells within the bladder wall. (a) N-cadherin+ cells with multiple processes in the lamina propria. (b) Closely associated N-cadherin+ cells at the boundary of smooth muscle bundles. Note large size and stellate morphology. (c) N-cadherin+ cells running between smooth muscle fascicles show elongated instead of stellate morphology and reveal lateral branches (arrow) running into the muscle fascicles. (d) Intrafascicular, slender elongated N-cadherin+ branches run in parallel with the smooth muscle bundles. Magnification ×400. Uro: urothelium; Det: detrusor muscles.

**Figure 2:**
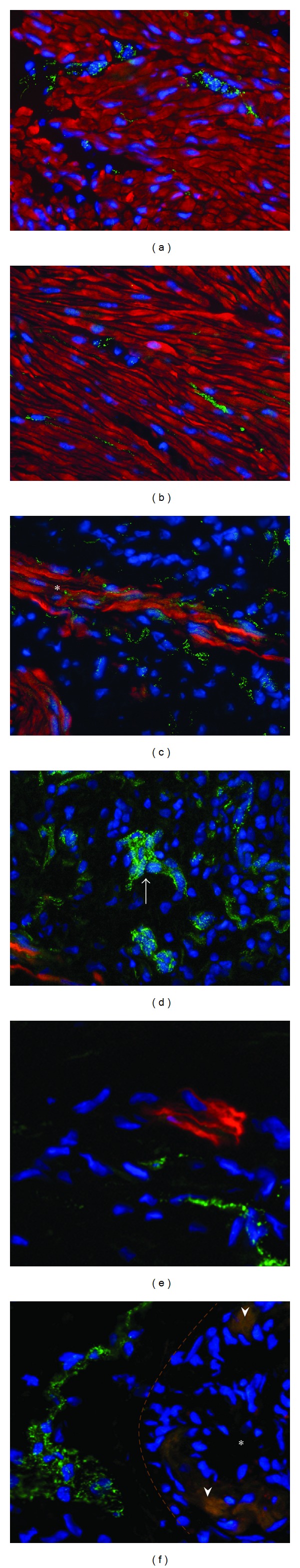
Double staining of N-cadherin with smoothelin and PGP9.5. (a–d) Costaining of N-cadherin (green) and smoothelin (red) in the bladder wall shows no colocalization. (a) Transversal and (b) longitudinal sections. (c) N-cadherin+ structures intermingle with smooth muscle cells in the region of the muscularis mucosae (asterix) (d) Note the aggregation of multiple N-cadherin+ cells in the lamina propria (arrow). (e) PGP9.5 is expressed by a nerve trunk (red). (f) Aggregated N-cadherin+/PGP9.5− cells are closely associated with a ganglion region (asterisk). Ganglion coexpressesbackground signal of N-cadherin, resulting in orange bodies (arrowheads). Note high contrast to the punctate expression of N-cadherin in the N-cadherin+/PGP9.5− cells. DAPI (blue) for nuclei. Magnification ×400.

Throughout the entire bladder wall, N-cadherin positive structures were found. These structures were located immediately below the urothelium, throughout the lamina propria and in the detrusor layer (Figure 1). Counterstaining with DAPI showed that the N-cadherin+ structures embodied branched cells provided with multiple processes (Figure 2). N-cadherin expression showed a punctate pattern distributed throughout the entire cell body.

Suburothelial N-cadherin+ cells had branched morphology with multiple processes that seemed to form a network. In the detrusor, N-cadherin+ cells were found at different levels. N-cadherin+ cells with stellate morphology were also located at the boundaries of smooth muscle bundles. They seemed to interact with elongated N-cadherin+ cells running in the interfascicular planes, continuing as slender N-cadherin+ processes between smooth muscle cells.

### 3.2. Phenotyping of N-Cadherin Positive Cells

Staining for smoothelin confirmed that N-cadherin+ but smoothelin-cells were housed at the border of smooth muscle fascicles (Figure 2). Inside the fascicles, they continued as elongated processes running in parallel with smooth muscle orientation spanning numerous smooth muscle cells. Irregularly arranged bundles of cells expressing smoothelin were found midway between the urothelium and the detrusor smooth muscle bundles. Those so-called muscularis mucosae varied considerably in diameter and formed a discontinuous layer of cells, densely surrounded and traversed by N-cadherin+ structures.

PGP9.5 immunoreactivity was found in both the lamina propria and detrusor layer. In the detrusor layer, primary nerve trunks run close to the detrusor bundles. Secondary neuronal structures were found in the connective tissue between smooth muscle fascicles, whereas smaller fibers run between small groups of smooth muscle cells. Double staining of N-cadherin and PGP9.5 showed no colocalization. However, close association between N-cadherin+ cells and PGP9.5+ neuronal structures was found (Figure 2).

**Figure 3:**
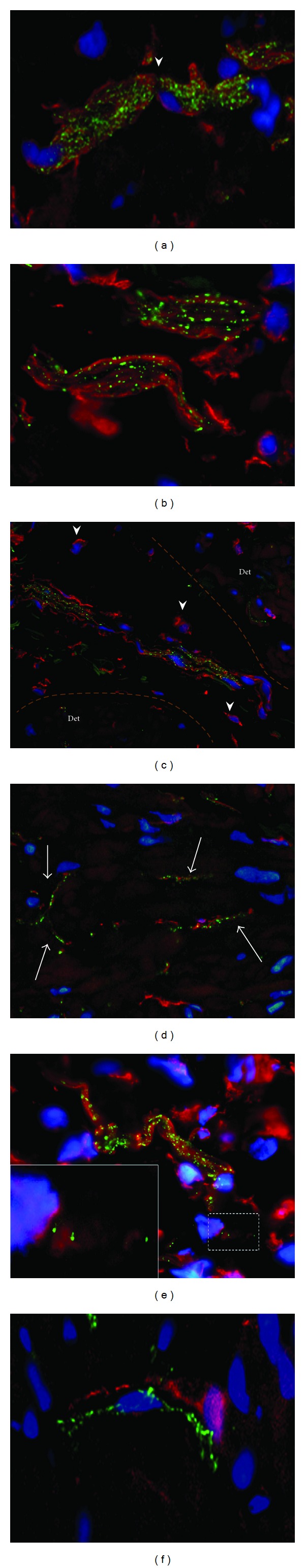
Double staining of N-cadherin and vimentin. (a) Two suburothelial cells coexpressing N-cadherin (green) and vimentin (red) are closely associated with each other (arrowhead). (b) Note punctate pattern of N-cadherin in contrast to the vimentin+ filaments, which both are expressed within the same cell body. Magnification ×1000. (c) Multiple elongated N-cadherin+/vimentin+ containing cells are closely associated at the border of detrusor smooth muscle bundles. (d) Detrusor fascicles show numerous N-cadherin+/vimentin+ processes (arrows) running between muscle cells. Magnification ×630. (e) Higher magnification shows expression of N-cadherin that is located at the cell membrane of some vimentin+ cells. Magnification ×1000. (f) Two cells localized at the edge of a smooth muscle fascicle. N-cadherin+/vimentin− cell is neighboured by a N-cadherin−/vimentin+ cell. Magnification ×1000.

N-cadherin and vimentin were coexpressed by cells of ramified morphology in the suburothelial layer and deeper lamina propria (Figure 3). These cells seemed to form a suburothelial network. However, many vimentin+ cells did not express N-cadherin. These N-cadherin−/vimentin+ cells showed different morphology. They were smaller and less elongated, had little perinuclear cytoplasm, and presumably embodied fibroblasts.

N-cadherin+/vimentin+ cells were also found at the border of detrusor smooth muscle bundles. Similar to the suburothelial region, these cells seemed to interconnect with each other, expanding into and throughout the smooth muscle fascicles like a network of N-cadherin+/vimentin+ processes.

The punctuate pattern of N-cadherin expression was expressed throughout the entire cell body and at the cell membrane. In general, the small vimentin+ cells with little perinuclear cytoplasm did not express N-cadherin.

Most N-cadherin+ cells coexpressed vimentin. However, a second type of N-cadherin+ cells was found that did not coexpress vimentin. These cells were not housed between smooth muscle cells but were restricted to the edge of smooth muscle fascicles and were also found in the lamina propria (Figure 3(f)). In contrast to the N-cadherin+/vimentin+ cells, these N-cadherin+/vimentin− cells showed different morphology: they had small appearance with little perinuclear cytoplasm sprouting into multiple cytoplasmatic processes.

### 3.3. N-Cadherin Positive Cells and Interstitial Cell Marker C-Kit

**Figure 4:**
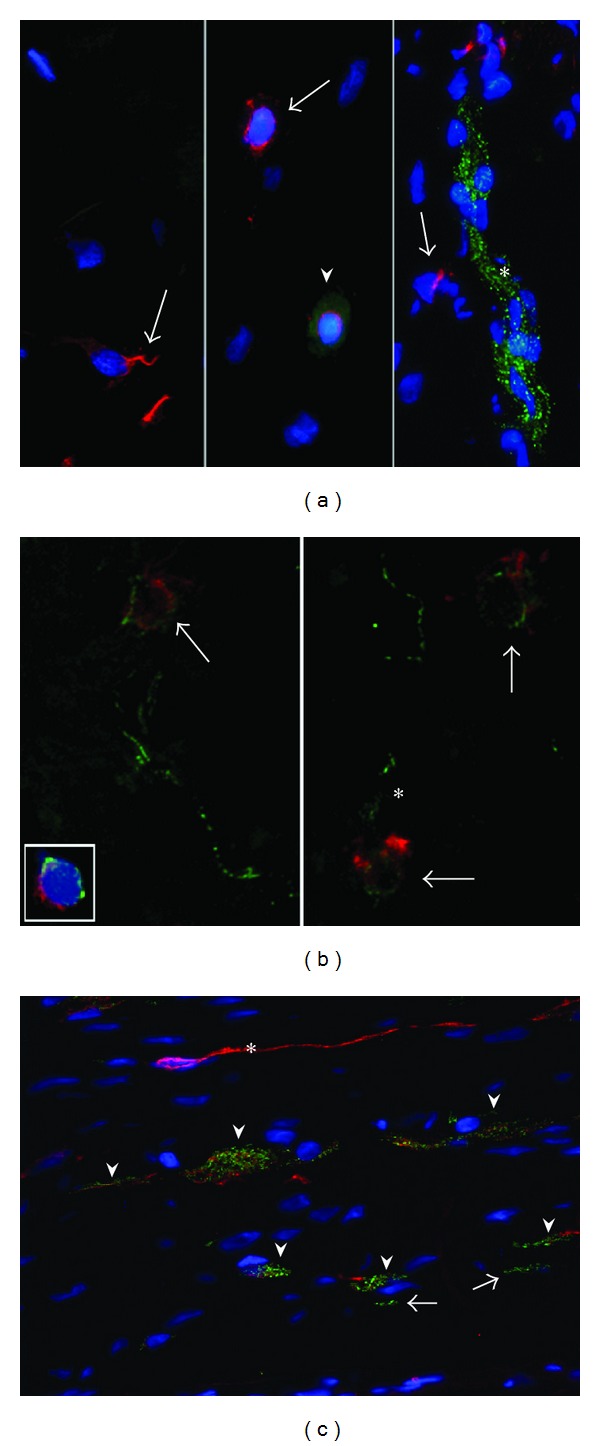
Double staining of N-cadherin and C-kit. (a) Arrows show cells in the bladder expressing C-kit (arrows, red). They lack expression of N-cadherin (green). Arrowhead shows a round cell with perinuclear expression of C-kit and diffuse cytoplasmic background signal for N-cadherin, highly resembling a mast cell. Elongated clusters of punctate N-cadherin+ cells lack expression of C-kit (asterisk). Magnification ×630. (b) Staining lacking DAPI for better orientation. Arrows show cells in the bladder expressing C-kit and punctate N-cadherin. These cells seem to give rise to N-cadherin+ branches (asterisk). Magnification ×1000. (c) N-cadherin+/C-kit+ cells running parallely (arrowheads) are neighboured by a slender elongated N-cadherin−/C-kit+ cell body (asterisk) and several smaller N-cadherin+/C-kit− cells (arrows) in the jejunum. Magnification ×630.

C-kit+ cells were found throughout the entire bladder wall (Figure 4). A large number of these cells coexpressed N-cadherin and showed similar morphology to the previously described N-cadherin+/vimentin− cells (Figure 4(b)). They were located on the boundary of smooth muscle fascicles and in the lamina propria.

Specimens of the human jejunum were used as a positive control for C-kit. The gut showed a large population of cells coexpressing N-cadherin and C-kit. Although most cells showed coexpression of N-cadherin and C-kit, N-cadherin+/C-kit− and N-cadherin−/C-kit+ cells were also found.

### 3.4. Ultrastructure of ICs in Lamina Propria and Detrusor Muscle

**Figure 5:**
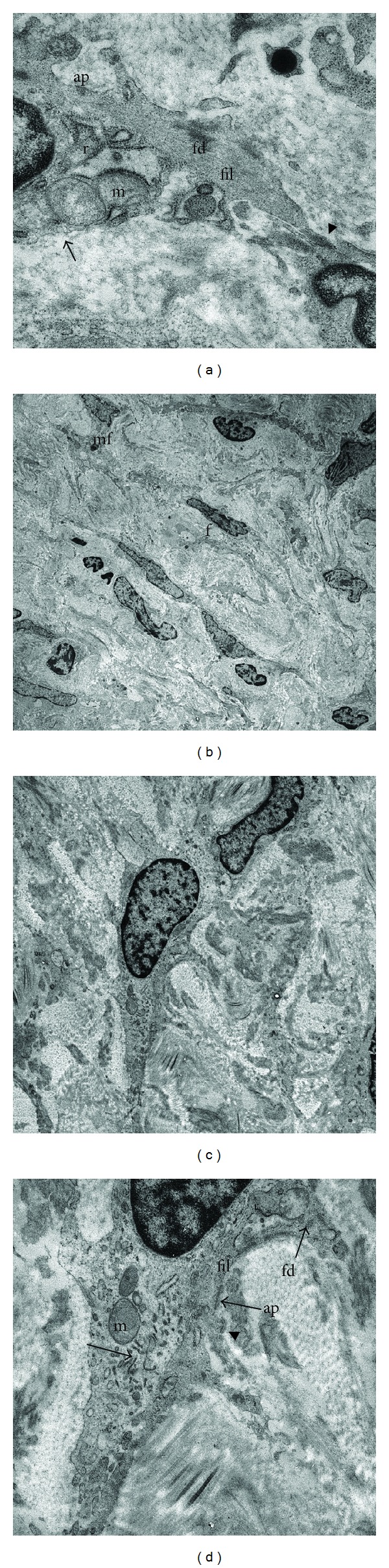
Ultrastructure of interstitial cells. (a) A myofibroblast located in the lamina propria. The cell is identified by filaments (fil), focal densities (fd), membranous attachment plaques (ap), subsurface vacuoles (arrow), mitochondria (m), and prominent rough endoplasmic reticulum (r). Cytoplasmic filaments cohere to a membranous attachment plaque, showing intimate association with its neighboring cell (arrowhead). Magnification ×420,000. (b) Overview of cells in the lamina propria. The left upper corner shows a myofibroblast (mf). Note its stellate morphology with multiple branches. The cell is accompanied by fibroblasts (f). Magnification ×10,500. (c) Two closely associated interstitial cells in the detrusor layer. (d) Higher magnification of (c). Branched interstitial cell shows mitochondria (m), interrupted basal lamina (arrowhead), peripheral filaments (fil), membranous attachment plaques (ap), focal densities (fd), and prominent rough endoplasmic reticulum (r). Numerous tubulovesicular structures (open arrow) were found exclusively in this cell type.

In electron microscopy, interstitial cells with stellate morphology were found in the lamina propria and the musculus detrusor layer. They had characteristic features of myofibroblasts, such as cytoplasmatic filaments, focal densities and membranous attachment plaques, interrupted basal lamina of extracellular matrix, numerous mitochondria, and prominent rough endoplasmic reticulum (Figure 5). From a morphological point of view, they appear to be similar to the N-cadherin+ cells as described in Figure 1.

## 4. Discussion

During the filling phase, the human bladder shows contractile activity which is generated within the bladder wall and does not result in intravesical pressure rise. It is also called autonomous activity [1–3]. It has been proposed that this activity shares characteristics with electrical rhythmicity in gastrointestinal muscles. Throughout the gastrointestinal tract, a network of interstitial cells of Cajal (ICCs) acts as pacemakers and conductors of electrical activity along the gut wall [5]. Initially, interstitial cells (ICs) were thought to represent a specialized type of neurons, but it is now concluded that they are a unique class of cells [6]. As ICs are also found in the human bladder, it has been proposed that they mediate autonomous bladder activity [2].

Currently, ICs are identified by various surrogate phenotypic markers, but no immunophenotype has been identified that is fully characteristic for ICs in the human bladder [9]. Irrefutable confirmation of the interstitial phenotype depends on application of electron microscopy, which is highly time consuming. This study was performed in search of a specific marker for interstitial cells in the human bladder and electron microscopic investigations of the same biopsies had to confirm the immunohistochemical findings.

It has been suggested that ICs of the human bladder form a network of interacting cells [7, 8]. Cadherin complexes participate in cell-cell recognition by functioning as signaling centers [10, 11]. The subtype N-cadherin is known to regulate mesenchymal cell development [13] and is the most commonly expressed cadherin in the interstitial compartment [14, 15]. We therefore investigated the expression of N-cadherin in the human normal bladder.

N-cadherin+ cells were found in the lamina propria and the detrusor layer. They showed abundant punctuate expression of N-cadherin at their cell membrane and throughout their cell body. We cannot fully explain why N-cadherin was not exclusively expressed at the plasma membrane. However, other investigators also found that cadherins can be localized intracellular, rather than being characteristically concentrated at regions of cell-cell contact [18].

Additional cell markers were used to further analyze our findings. Smoothelin is a smooth muscle cell specific marker [16]. As no colocalization of N-cadherin and smoothelin was found, we believe that the N-cadherin+ cells do not represent smooth muscle cells.

Vimentin is expressed by fibroblastic cells [9]. A large population of N-cadherin+ cells coexpressed vimentin. They showed elongated or stellate morphology with multiple processes that seemed to form a network. However, many vimentin+ cells did not express N-cadherin. These cells were smaller and less elongated, had little perinuclear cytoplasm compared to the N-cadherin+/vimentin+ cell, and appeared to be regular fibroblasts.

PGP9.5 was chosen as a pan-neuronal marker as it is generally accepted that this protein is expressed by all neuronal structures of the bladder wall [19]. No colocalization of N-cadherin+/vimentin+ cells and PGP9.5 was found. However, N-cadherin+ cells and PGP9.5+ neurons were closely associated. Furthermore, as mature neurons lack expression of vimentin [20], we believe that the N-cadherin+/vimentin+ cells do not represent neuronal structures.

C-kit is a widely used marker for ICCs of the gut [21]. We used specimens of the human gut as a positive control. ICCs of the gut coexpressed N-cadherin and C-kit. In the bladder, N-cadherin and C-kit were coexpressed by cells with little perinuclear cytoplasm seeming to sprout into N-cadherin+ processes. As these cells seemed highly similar to C-kit+ cells in human detrusor as found by others [22], we believe that they embody interstitial cells.

Interstitial cells are found in both the lamina propria and the detrusor layer of the human bladder. Myofibroblasts are a recently documented interstitial cell type housed in the interstitial compartment. They share characteristics with smooth muscle cells and fibroblasts. In a previous study, no ultrastructural evidence for myofibroblast differentiation in the detrusor layer was discerned [23]. Interstitial cells within this layer were identified as fibroblasts. It is therefore generally believed that myofibroblasts in the human bladder solely refer to a specific group of interstitial cells within the suburothelial layer [24]. However, during our study, TEM revealed interstitial cells with stellate morphology in the lamina propria and the musculus detrusor layer. They had characteristic features of myofibroblasts. We previously showed that a suburothelial layer of cells expresses alpha smooth muscle actin myofilaments [12], most probably embodying the cytoplasmic filaments as shown during TEM in this study. Furthermore, fully differentiated myofibroblasts express alpha smooth muscle actin [25]. We therefore conclude that both the lamina propria and the detrusor layer house myofibroblasts, a unique class of interstitial cells.

From a morphological point of view, ultrastructurally defined myofibroblasts in the lamina propria and detrusor layer appear to be similar to the N-cadherin+/vimentin+ cells. Both techniques identified specified cells of mesenchymal origin with highly branched morphology and multiple processes that were closely associated with neighbouring homotypic cells.

This study shows a population of cells from mesenchymal origin with multiple phenotypes, immunopositive for N-cadherin, vimentin, and C-kit. These cells are housed in the interstitial compartment throughout the entire human bladder wall. The findings are in accordance with a recent study of Monaghan et al. in which multiple subgroups of vimentin+ cells with distinctive morphology were found in all layers of the bladder wall [26]. It is therefore likely that not all IC's may be labeled with the same markers.

Heterogeneity of the interstitial compartment could be explained by a model in which C-kit and N-cadherin regulate mesenchymal cell differentiation. C-kit is an important stem cell marker used to identify certain types of progenitor cells [27]. Signaling through C-kit plays a role in cell differentiation, proliferation, and survival. The cadherins constitute a superfamily of glycoproteins that participate in cell-cell recognition [10]. Like C-kit, they play a crucial role in cellular differentiation and embryogenesis.

From a functional point of view, one should consider the following characteristics associated with the markers used. Similar to the gut, C-kit+ cells in the human bladder possibly act as pacemaker cells from which spontaneous calcium transients originate [28]. Cadherin complexes play a major role in cell-cell recognition and function as signaling centers [11]. Therefore, the population of N-cadherin+/vimentin+ cells may participate in specialized events such as spread of pacemaking activity. Although our results are promising, this study relies on morphological evidence. In order to illuminate functional properties of these cells, future research is needed using functional cell analyses, such as Ca-imaging and patch clamp techniques.

**Figure 6:**
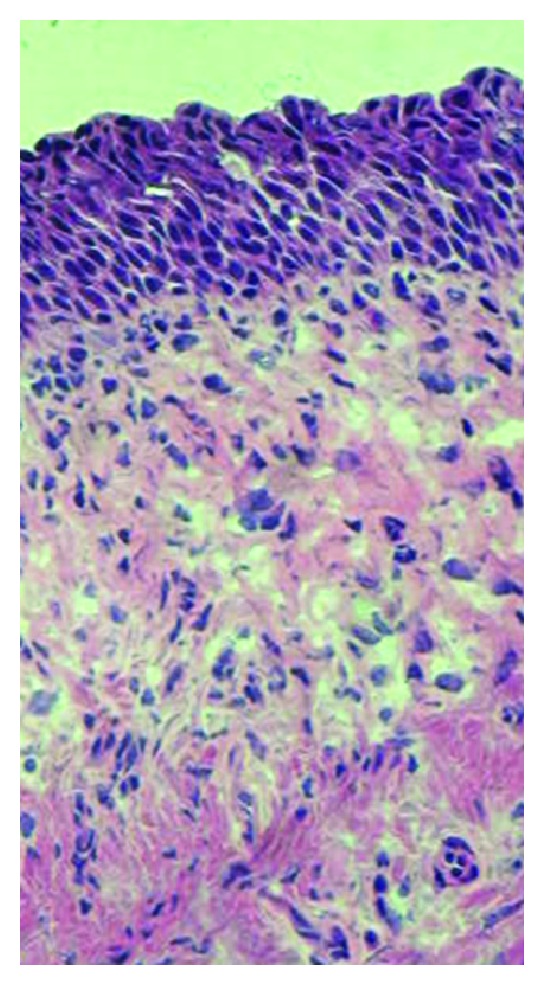
Representative hematoxylin and eosin stain of urothelial area of the specimens used. Normal urothelium consists of approximately 3–5 cell layers. Note that no abnormal thickening of the urothelial layer or abnormal suburothelial morphology is found.

Although dissection of our specimens was performed distant from tumor sites, an influence of cancer cannot be ruled out. However, no thickening of the urothelial layer or abnormal urothelial morphology was found (Figure 6). Also, urothelium did not express N-cadherin, which is often seen in urothelial bladder cancer [29]. We therefore believe that our findings are unaffected by tumor-related factors.

## 5. Conclusions

This study shows that the interstitial compartment of the human bladder houses a heterogenous population of cells from mesenchymal origin, immunopositive for N-cadherin, vimentin, and C-kit. Due to characteristics associated with these proteins, this population of cells may participate in specialized events of the human bladder. We assume that N-cadherin/vimentin is a specific marker for a subpopulation of interstitial cells in the human bladder, that is, the ultrastructurally defined myofibroblasts. These cells may participate in spread of pacemaking activity. Although further insight is needed in the correlation between morphology and function of these cells, these findings could be promising in understanding normal and overactive bladder behaviour. Furthermore, we question the possible existence of one specific marker which defines the entire group of ICs in the human bladder.
